# The landscape of lung cancer screening globally: a comparison of established programs and estimates of eligibility to participate

**DOI:** 10.1016/j.lanwpc.2026.101862

**Published:** 2026-04-22

**Authors:** Hannah Jongebloed, Lan Gao, Hua-Hie Yong, Patricia M. Livingston, Victoria M. White, Coral E. Gartner, Geoffrey T. Fong, Andrew Hyland, Katherine A. East, Krzysztof Przewozniak, Hong Gwan Seo, Yeol Kim, Vivienne Milch, Anna Ugalde

**Affiliations:** a Institute for Health Transformation, Centre for Quality and Patient Safety Research, School of Nursing and Midwifery, Faculty of Health, Deakin University, Geelong, Victoria, Australia; b Deakin Health Economics, Institute of Health Transformation, Faculty of Health, Deakin University, Geelong, Victoria, Australia; c School of Psychology, Deakin University, Geelong, Victoria, Australia; d Faculty of Health, Deakin University, Geelong, Victoria, Australia; e NHMRC Centre of Research Excellence on Achieving the Tobacco Endgame, School of Public Health, The University of Queensland, Herston, Queensland, Australia; f Department of Psychology, University of Waterloo, Waterloo, Ontario, Canada; g School of Public Health Sciences, University of Waterloo, Waterloo, Ontario, Canada; h Ontario Institute for Cancer Research, Toronto, Ontario, Canada; i Department of Health Behavior, Roswell Park Comprehensive Cancer Center, Buffalo, NY, USA; j Department of Primary Care and Public Health, Brighton and Sussex Medical School, University of Brighton and University of Sussex, Brighton, Sussex, UK; k Department of Addictions, Institute of Psychiatry, Psychology and Neuroscience, King’s College London, London, UK; l Global Institute of Family Health, University of Kalisz, Kalisz, Poland; m Global Research, Implementation and Training Lab, Department of Population and Public Health Sciences, Keck School of Medicine, University of Southern California, Los Angeles, CA, USA; n Department of Disease Screening, Design Hospital, Jeonju-si, Jeonbuk-do, Republic of Korea; o Department of Family Medicine, National Cancer Center, Goyang-si, Republic of Korea; p Graduate School of Cancer Science and Policy, National Cancer Center, Goyang-si, Republic of Korea; q Caring Futures Institute, College of Nursing and Health Sciences, Flinders University, Adelaide, South Australia, Australia

**Keywords:** Lung cancer screening, International comparison, Policy audit, Cancer screening, Screening eligibility, Early detection of cancer, Mass screening, Health equity

## Abstract

Comparing lung cancer screening programs worldwide provides insight into the most utilised screening approaches and variations in program eligibility. This study compared the eligibility criteria and parameters for lung cancer screening programs internationally and modelled the reach of each program. Eleven countries with implemented, organised, national or regional lung cancer screening programs were identified; eight (73%) utilised pack-years to measure smoking intensity, with four countries adopting 30-pack-years as a requirement. Three programs (27%) utilised risk prediction models to determine eligibility. Proportions of eligible people who currently smoke range from 10.7% to 27.8%, with the fewest in the Republic of Korea (10.7%) and most in the United States (27.8%). Globally, lung cancer screening eligibility criteria and program parameters vary substantially. There was limited consideration for engaging key population groups. Ensuring programs incorporate relevant local strategies to maximise participation across at-risk groups is essential, especially for those underrepresented in screening programs.

## Introduction

Globally, lung cancer is the most commonly diagnosed cancer and the leading cause of cancer-related death.^1^ In 2020, an estimated 1.8 million deaths worldwide were attributable to lung cancer.^2^ Lung cancer is often diagnosed at a late stage, when treatment options are limited and ineffective, leading to poorer survival outcomes compared to many other malignancies.^3^ Prevention of lung cancer is primarily focused on reducing behavioural risk factors, including tobacco control measures and environmental risk factors such as occupational exposure to chemicals and asbestos.^3^

Screening for lung cancer is now an early detection strategy with increasing international adoption, reflecting the growing evidence of its survival benefits. International trials have demonstrated the effectiveness of screening for lung cancer in reducing the number of deaths by identifying cases of lung cancer at earlier stages.^4^, ^5^, ^6^ Unlike population-based cervical, breast and bowel cancer screening programs, lung cancer screening is risk-based, targeting people with a high risk of lung cancer to undergo a low dose computed tomography (LDCT) scan.^7^ While several countries are implementing lung cancer screening,^8^^,^^9^ some countries are yet to adopt this program, citing feasibility concerns and radiation exposure for participants.^8^

For screening to be successful and effective, screening programs require sufficient uptake by those eligible to participate.^10^ There is evidence of the disproportionate impact of lung cancer on certain population groups.^11^^,^^12^ Disparities in participation rates are also often observed between these population groups and the broader screening-eligible population. Culturally diverse communities, and people from low socio-economic groups, rural areas, and Indigenous communities can have lower engagement with lung cancer screening programs.^13^^,^^14^ This evidence indicates a need to focus efforts to reduce lung cancer disparities and improve timely, equitable treatment.

There are known challenges in engaging eligible participants in lung cancer screening programs. In the United States (US), where LDCT screening has been formally recommended since 2015, participation rates were low at 3.3% in 2015, increasing to 18% in 2022.^15^^,^^16^ In the Republic of Korea, participation rates have been reported at 53%, having increased from 33% in 2019.^17^ Adherence to lung cancer screening is known to decline following the initial baseline scan^18^ and is lower than that observed in large randomised trials, with people who currently smoke less likely to adhere to screening intervals compared with those who have previously smoked.^19^

The implementation and structure of lung cancer screening programs vary internationally.^8^ Several studies have described existing lung cancer screening programs, focusing on differing points of comparison including the histories of program establishment and application of risk stratification models. However, there is opportunity to build upon comparison of programs, to explore the impact of eligibility criteria on engagement with the program.^8^^,^^20^ As countries establish, evaluate, and modify lung cancer screening programs, and given the known challenges in engaging eligible participants, identifying best practices and contextual factors within existing programs is important to maximising implementation and adoption. This study aimed to compare across countries, the program parameters, engagement strategies, eligibility criteria and estimated reach of lung cancer screening programs to estimate the proportion of the population eligible to participate.

## Methods

To address the study aims, two forms of analysis were undertaken: 1) a policy landscape analysis^8^ of existing lung cancer screening programs; and 2) estimating the eligible population size of each country’s screening program. The first analysis involved identifying countries that have a current national/regional screening program meeting the inclusion criteria, and sourcing relevant policy documents. The second involved utilising relevant country-specific data on population and tobacco use statistics supplemented by smoking-related data available from countries that participated in the International Tobacco Control (ITC) Policy Evaluation Project (Australia, Canada, US, England, Poland, and Republic of Korea) to estimate the screening-eligible population size for each program.

### Policy document analysis

#### Inclusion criteria

Screening programs were considered eligible for inclusion if they met the criteria of an active national or regional program as defined by the Lung Cancer Policy Network (2nd Ed.),^21^ which are defined as formally endorsed and organised national or regional lung cancer screening programs utilising LDCT.^22^ While several countries are exploring lung cancer screening programs; this study focused on established, endorsed programs, where the criteria are confirmed and required data are available. Screening programs were ineligible if they were in pilot phase, were part of a trial, or utilised screening models that prohibit comparison (i.e. programs that are solely mobile screening).

#### Search strategy

Countries were identified from the Lung Cancer Policy Network map in March 2025,^21^ and assessed against the inclusion criteria. A search for country-specific documents including national guidelines, position statements and policy documents describing lung cancer screening programs was undertaken for each country meeting the inclusion criteria. Documents were identified for each country via the internet by targeting websites of relevant government departments and organisations. Where data were unable to be retrieved, relevant contacts listed on a country’s primary resource were contacted for assistance with obtaining further information. Where no further information was available, or making contact was unsuccessful, country-specific grey literature sources were supplemented by peer-reviewed academic literature. Searches were conducted in English and where key resources were published in a language other than English, they were translated into English using Google Translate^23^ for the purpose of data extraction.

#### Data extraction

From documentation sourced for programs meeting the inclusion criteria, data were extracted on: date of program commencement, eligibility criteria for populations targeted for screening, the entry pathway to the program, key population groups identified for each program, strategies to target key population groups, description of the screening program (including frequency of scans and follow up), workforces involved with the program, changes to the program since inception, and evidence or plans to evaluate the program.

#### Data analysis

A narrative synthesis was conducted to report on the parameters of each program obtained during data extraction, consistent with past policy landscape analyses.^9^

### Screening-eligibility estimates

The population eligible for screening for each program was determined by utilising data on total population size, percentage of the population within each of the programs’ eligible age ranges and relevant smoking statistics. These were used to estimate the percentage of each country’s population that are eligible to participate in their screening program, by applying their respective eligibility criteria.

#### Population size and age data

Population data for the year 2022, stratified by 1-year age groups and sex were sourced from the 2024 Revision of World Population Prospects published by the United Nations.^24^ Region-specific population estimates for British Columbia and Ontario were obtained from the national statistical agency of Canada.^25^ England-specific population estimates were extracted from the Office for National Statistics.^26^

#### Current smoking rates and tobacco usage data

Tobacco usage data were accessed from the ITC Project. The ITC Project is an international cohort study, which measured the factors associated with tobacco use, including evaluation of the impact of tobacco control policies on smoking related beliefs, attitudes, and behaviours.^27^^,^^28^ The most recent available tobacco usage data were used to compute a measure of pack-years for each person who was identified as currently smoking in the sample. Data analysed came from the 2022 ITC Four Country Smoking and Vaping Survey (4CV) for Australia, Canada, England, and the US,^27^ the 2018 ITC 6 European (6 E) Country Project for Poland,^29^ and the 2024 ITC Korea (KRA) Survey.^30^ The detailed methods for these surveys have been described elsewhere.^31^, ^32^, ^33^ Estimates calculated using the Polish ITC data were used as a proxy for Croatia and Czechia, given the similar regional characteristics and higher smoking rates than those observed in the European Union.^34^^,^^35^

Current smoking rates were obtained from The Global Health Observatory Tobacco control estimates of current cigarette smoking prevalence^36^ for each country included in the modelling, with the exception of England. England-specific estimates were extracted from the Office for National Statistics Adult Smoking Habits in the UK: 2023 release.^37^

#### Risk prediction tool data

Estimates of the proportion of the assessed population meeting the risk assessment thresholds for countries utilising a risk assessment tool for eligibility were obtained from the published peer-reviewed literature: Prostate, Lung, Colorectal, and Ovarian cancer screening trial model 2012 (PLCOm2012) risk of ≥1.51% over six years or Liverpool Lung Project risk model version 2 (LLPv2) five-year risk of ≥2.5% (England),^38^ PLCOm2012 risk of ≥1.5% over 6 years (British Columbia; Canada),^39^ and PLCOm2012 risk of ≥2% over 6 years (Ontario; Canada).^40^

#### Analysis

Three variables were established to inform analysis:

• *Years smoked;* it was assumed that smoking commenced at age 18^41^ and smoking duration and age at the time of the survey was calculated to estimate the number of years a respondent had been smoking.
• *Number of packs per day;* Average number of cigarettes per day (CPD) divided by 20 (the standard cigarette pack size used in pack year calculations), to estimate the number of packs a respondent smoked per day.
• *Pack-years;* Estimated by multiplying the number of years a respondent had smoked by the number of packs per day, creating an estimate of all respondents who identified as currently smoking within the sample. Pack-years was then coded as 1 (0–9.99 pack-years), 2 (10–19.99 pack-years), 3 (20–29.99 pack-years) and 4 (30 and above pack-years). The distribution of responses across these categories was estimated using weighted data to correct for any sampling biases.

To enable cross-country comparisons, two estimates were calculated for a single year (2022): (1) the proportion of people who currently smoke that are eligible to participate in each country, and (2) the proportion of the total adult population represented by these eligible people who currently smoke. These were established by identifying the number of people who currently smoke within the applicable age range (country dependant) and applying the proportion meeting the pack-year or risk assessment thresholds, which was established via the ITC datasets, or where programs utilised a risk assessment tool to assess eligibility, sourced from the appropriate peer-reviewed literature where required (Canada & England).

IBM SPSS v30^42^ was used to compute a pack-years variable using computed and existing variables in the ITC dataset. Microsoft Excel^43^ was used to model the eligible screening population utilising population size and age data, current smoking rates and tobacco usage data, and risk assessment data. Estimates of the eligible proportion of the population and 95% confidence intervals were computed using a normal approximation method.^44^

### Ethical approval

An ethics exemption was granted by the Deakin University Human Research Ethics Committee for the secondary analysis of de-identified ITC data.

## Results

Overall, 11 lung cancer screening programs were identified as meeting the inclusion criteria. These include three regional programs: British Columbia (Canada), Ontario (Canada), and Emirate of Abu Dhabi (United Arab Emirates [UAE]), and eight national programs: Croatia, Czechia, Poland, the Republic of Korea, Taiwan, the US, Australia and England.

### Key parameters of lung cancer screening programs

Internationally, lung cancer screening programs are highly variable. For most programs (n = 8, 73%), entry required support or facilitation from a primary healthcare professional (Australia, British Columbia, Croatia, Czechia, England, Ontario, Taiwan, US). Other entry models included centralised telephone services (n = 2, 18% [British Columbia and Ontario]). Common exit pathways included reaching the upper age limit of the program, and the development of health problems which prohibited the individual from participating in screening or from having curative treatment. Six programs (55%) have an annual screening interval, with the remaining programs recalling participants with a negative scan on a biennial basis (Australia, British Columbia, Croatia, England, Taiwan). The Lung CT Screening Reporting & Data System (Lung-RADs) assessment categories for classification of nodules was the most utilised (n = 6, 55%). However other named nodule management protocols included the Pan-Canadian Early Detection of Lung Cancer Study (PanCan), the British Thoracic Society (BTS) 2015 pulmonary nodule guidelines and the National Institute for Health and Care Excellence (NICE) guidelines for the management of lung cancer. Seven programs (64%) did not identify key population groups to target for inclusion in lung cancer screening. Six programs (55%) described a mechanism for evaluating their program. The importance of smoking cessation was outlined in all program guidelines and policy documents, with several programs detailing specific smoking cessation supports that were incorporated into their screening program (British Columbia, Croatia, Emirate of Abu Dhabi, England, Poland). The parameters of each included country’s programs are provided in Table 1.

**Table 1:** Lung cancer screening program parameters.

Country and program name	Date^a^	Entry pathway	Exit pathway	Screening interval	Nodule classification	Key population groups recognised within the programs	Evaluation of the program	Smoking cessation delivered within the program
Australia: National Lung Cancer Screening Program^45^^,^^46^	2025	New participants may enter the program via one of the following pathways. All pathways include an eligibility assessment performed by a primary health care professional: • Self-identification–proactively requests eligibility assessment, • Facilitated entry by a primary care professional, • Opportunistic entry–assessment is offered during a consultation that was arranged for another purpose, • Organised entry–potential participants are identified and invited to participate in an eligibility assessment, • Facilitated entry–support for potential participants to arrange a primary care appointment. May include engaging a support person such as a family member or community worker to support them.	• Passing the upper age limit. • Identification of a high-risk or very high-risk nodule.	Biennial	Nodule management protocol which incorporates: • Pan Can nodule management protocol for baseline scan, • Modified Lung-RADS assessment categories at follow-up.	• Aboriginal and/or Torres Strait Islander people. • People living in rural and remote areas. • People from culturally and linguistically diverse (CALD) backgrounds. • People with disability. • People with mental illness. • People from the Lesbian, Gay, Bisexual, Transgender, Intersex, Queer and Asexual (LGBTIQA+) communities.	• A program Quality framework which includes performance indicators and quality standards. • Biopsy and adverse event form included to monitor adverse events from screening participation. • Program evaluation is to be undertaken two years after program commencement.	Healthcare providers are expected to offer smoking cessation support through existing infrastructure.
Canada: British Columbia Lung Screen Program^47^	2022	• Identification of at-risk patients and referral by Primary care provider for screening. • Risk assessment and patient intake is centralised through a provincial call centre. • PCPs can fax referral forms for patients who may experience barriers to self-referral. • Self-referral also accepted (must have PCP)	• Reach upper age limit. • Development of health problems that substantially limits life expectancy or the ability or willingness to have curative treatment.	Biennial	• Pan Can Lung nodule management protocol for the baseline LDCT. • Volumetric protocol when two or more LDCTs are available following BC Lung Screening Protocol version 1.	• First nations, Inuit and Métis. • Underserved populations (Racial or ethnic minorities). • Rural and remote populations.	Evaluation will occur on a regular basis and may include analysis of percentage of eligible individuals screened, screening appropriateness, quality assurance measures, adherence to management recommendations, return to regular screening, cancer detection rates, invasive procedures for benign disease, complications following biopsies or surgery, unplanned hospitalisations and deaths in 30 days.	Screening program to provide brief smoking cessation counselling over the phone, provision of educational materials, and optional engagement with ongoing counselling support and nicotine replacement therapy.
Canada: Ontario Lung Screening Program^48^^,^^49^	2021	• Referral from a healthcare provider to the Ontario Lung Screening Program (OLSP), or self-presentation to the OLSP where the site assesses against referral inclusion criteria. • Risk assessment call is then completed by a Screening Navigator and the primary care provider is notified of the outcome. If the individual does not have a PCP, they are linked with one at this time as a PCP is required to participate in the program. • If referred by a PCP, a program form authorising a LDCT scan must be completed at the same time.	NR	Annual	Lung-RADS assessment categories utilised.	• First Nations, Inuit and Métis. • Underserved populations (Underserved populations overall, People without a primary care provider, Non-English speakers, Non-binary and gender diverse people and Under screened populations as a result of the COVID-19 pandemic). • Rural and remote populations.	NR	Referral to existing infrastructure by healthcare providers and organisations.
Croatia: National Programme for Early Detection of Lung Cancer^50^	2020	Identification and initial opportunistic screening of persons by general/family physicians who will have a comprehensive conversation with the person and present the limitations, risks and benefits of screening.	NR	Biennial	The interval for indeterminate findings according to the recommendation of a specialist in clinical radiology. For persons included in the National Program in whom the LDCT finding is with nodules, control the interval for LDCT will be determined according to I-ELCAP criteria and recommendations.	NR	• Monitoring indicators for after five years of program implementation are: a) coverage of the population–to achieve response in the National Lung Cancer Program after five year of program implementation of 50% among smokers who meet the inclusion criteria for which the panel is filled. b) reduce the total mortality from lung cancer by 20%. • For the purposes of analysing the success and usefulness of the program appropriate protocols and methods for collecting and monitoring data (register and/or records) will be implemented, which will include patient outcome data with a focus on total morbidity and survival of person included in the program. • Quality of processed data will be monitored and reported on once a year. • Monitoring of implementation of the National Program. • Provision of suggestions and opinions for improving the implementation of the National Program including changes in professional guidelines, changes in the list of centres and the proposal of implementation of other activities that are necessary for the complete implementation of the National Program.	Smoking cessation counselling is provided by the referrer for the LDCT. The No-Smoking school, which is organised by the Croatian Thoracic Society, the Croatian Institute of Public Health and the Public Health Institutes. Will be offered free of charge to all persons included in the National Program.
Czechia: Early Detection Programme for Lung Cancer^51^^,^^52^	2022	Individuals contact their GP who explains the program and makes a referral to an outpatient lung specialist. A pulmonary specialist takes a medical history and conducts a standard lung examination. Eligible patients will be referred to a local radiology department.	NR	Annual, moving to Biennial screening as per nodule management	• Negative results: schedule next CT scan on the recommended screening schedule (1–2 years). • Indeterminate: recommend repeat LDCT, usually between 6 and 8 week and 1 year. • Positive: arrange further investigations and consultation at a cancer centre. • Other findings: arrange further investigations.	NR	NR	Screening program combined with a smoking cessation program.
England: National Targeted Lung Cancer Screening Programme^38^	Planned roll out aiming for 40% coverage by March 2025, and 100% by March 2030	GP records used to identify current or former smokers. Risk is assessed based on smoking history and additional factors. Those identified as high risk invited to participate.	Passing the upper age limit.	Biennial	• BTS 2015 pulmonary nodule guidelines. • NICE guidelines for the management of lung cancer.	NR	Mechanisms detailing the evolution of the protocol included within the standard protocol including: • Evolution of the program over time, • Advice, support and guidance provided by the LCSP Expert Advisory Group, • Adaptations as further research findings from screening studies emerge, • Advice and consultation from the UK National Screening Committee (UKNSC).	Those who currently smoke are offered smoking cessation: Very Brief Advice (VBA) and formal smoking cessation service referral on an opt-out basis.
Poland: National Program of Early Lung Cancer Detection^53^	Planned for 2018, initiated 2020	There is a short test for individuals to check their eligibility. If they feel they are eligible, they can call and make an appointment. A qualifying visit takes place where eligibility is confirmed through an interview with medical staff regarding smoking history and health condition. If the person qualifies, they are referred for a LDCT. Protocol advocates for shared decision-making process between the person and healthcare professional.	• Person is no longer a candidate for surgical treatment (either due to their health or not consenting to possible surgical treatment).	Annual	Lung-RADS assessment categories utilised.	NR	The program will be subject to evaluation, after which it will be possible to continue it under Operational Programs co-financed by EU funds. The following performance measures are also used: number of LDCT scans conducted, percentage of people with radiological changes detected, percentage for whom no changes were detected and number of health care workers to partake in training. Additional monitoring and evaluation include: The number of people who have withdrawn from the program, the reasons for resigning from the benefits offered under the program, people’s satisfaction with participation in the program, the quality of educational services and the effectiveness of program promotion will be assessed using a survey. The following epidemiological measures will be captured: number of detected radiological lesions (cancerous and non-cancerous), number of detected lung cancers qualified for surgical treatment, assessment of changes in 3- and 5-year survival rates in patients covered by the program (at least after 10 years of program implementation), assessment of changes in the mortality rate for this group of cancers and assessment of the change in the mortality rate for this group of cancers.	Participation in the LDCT screening test constitutes a willingness to participate in anti-tobacco counselling available outside the program and active smokers undergo the following diagnostic tests during the screening: - a standardized interview regarding smoking intensity and dependence (Fagerström's test) - a motivation to quit smoking test (Schneider's test) These are to be carried out by a person trained in anti-tobacco counselling or coordinating staff after the person has been qualified to participate in the programme.
Republic of Korea: Korean National Lung Cancer Screening Program^54^	2019	• Subjects are selected, and health checkup sheets and notices on examination methods and procedures are sent to the selected subjects at the beginning of the year. • Smoking history and current smoking status are assessed based on a questionnaire from a general health checkup (including life transition period health checkup) within the two years prior to the relevant year. • Among participants in health insurance smoking cessation treatment, smoking history is confirmed through the questionnaire completed for project participation.	NR	Annual	Lung-RADS assessment categories utilised.	NR	NR	Actively encourage smoking cessation and use smoking cessation aids to help them quit. Patients should also be referred to existing infrastructure: government supported smoking cessation and quit-smoking support centres.
Taiwan: National Lung Cancer Screening Program^55^	2022	Eligible participants are required to contact a listed hospital to arrange a time for the LDCT.	NR	Biennial	• Eligible people with no abnormalities should return in 2 years (recommended that participants return to the same hospital for comparison of previous images to assist interpretation). • Cases suspected to be abnormal should be rescreened based upon physician recommendations (may be 3, 6 or 12 months depending on the results).	NR	NR	Requirement for those who participate and continue to smoke to agree to receiving smoking cessation services.
United Arab Emirates–Emirate of Abu Dhabi: Department of Health Lung Cancer Screening Program^56^	2018	• Opportunistic recruitment. • Booking an appointment online.	• Reaching the upper age limit (Turns 76 years old). • Has not smoked in 15 years. • Development of a health problem that leads to the patient being unable or unwilling to have surgery if lung cancer is found.	Annual	Lung-RADS assessment categories utilised.	NR	No formal description of an evaluation. Does include several performance indicators including participation rate, retention rate, abnormal recall rate, false positive rate, lung cancer detection rate, stage 1 lung cancer rate, incidental findings rate, tobacco quit rates among participants in the program and diagnostic interval.	Provision of tobacco cessation counselling and treatment for more than 10 min including brief advice, set up quitting date, offer pharmacological agents’ treatment, offer tobacco cessation specialist appointment and enforcement of maintaining tobacco abstinence if former tobacco user.
United States: National Lung Cancer Screening Program^57^	2013 (Initial USPSTF recommendation)	Risk is assessed based on age and calculation of smoking history (pack-years). If the person meets the eligibility criteria, a shared decision-making conversation should take place regarding choosing to participate in screening. Participants can enter screening through a range of pathways, referral from primary care being one.	• Participant has not smoked for 15 years. • Develops a health problem that substantially limits life expectancy or ability/willingness to undertake curative lung surgery.	Annual	Lung-RADS assessment categories utilised.	NR	NR	Smoking cessation consistent with the USPSTF recommendation counselling and interventions to prevent tobacco use and tobacco-caused disease should be provided.

Note. The language presented in this table is largely derived from, and consistent with, that from the policy documents referenced.

a Refers to the actual date or expected date of program commencement, NR–Not reported.

### Eligibility criteria for each program

Age ranges and smoking eligibility varied between programs (see Table 2). Across the programs, the youngest age to commence participation was 50 years, with five programs (45%) adopting this age (Australia, Croatia, Poland, Taiwan, US), and the oldest age of commencement was 55 years (n = 5, 45%). The majority of qualifying age groups covered a 20-year range, with the exception of the US, who had a 30-year eligible age bracket, with people ageing out of the program at 80 years of age. Australia’s program was the only one to cease screening after the age of 70 years, with all other programs continuing until a minimum age of 74 years.

**Table 2:** Program eligibility criteria.

Country/Program	Eligibility criteria for inclusion			Exclusion Criteria
	Age	Smoking/Risk	Other factors	
Australia: National Lung Cancer Screening Program^45^	50–70 years	• History of cigarette smoking of at least 30 pack-years; and if a former smoker, has quit within the past 10 years	• Asymptomatic	• Presents with symptoms indicative of lung cancer • Unsuitable for a low-dose CT and unable to complete a scan: ○ Weight exceeds restrictions of scanner ○ Unable to lie flat for a minimum of 5 min ○ Symptomatic lung infection ○ Full CT scan within the last 12 months, or planned within the next 3 months
Canada: British Columbia Lung Screen Program^47^	55–74 years	• Currently smoking or have smoked in the past (smoking history of 20 years +) • PLCOm2012 risk of ≥1.5% over 6 years	• Have a primary care provider • Asymptomatic	• Symptoms suspicious for lung cancer • Those with major co-morbidities such as severe chronic obstructive pulmonary disease, congestive heart failure, renal failure on dialysis • Previous lung cancer or other cancers on active treatment and follow-up • Weight exceeds CT scanner restrictions (>450 lbs) • Unable to lie flat and hold arms above the head for a CT scan
Canada: Ontario Lung Screening Program^48^^,^^49^	55–80 years	• Current and former smokers who have smoked cigarettes daily for at least 20 years (not necessarily 20 years in a row) • PLCOm2012 risk of ≥2% over 6 years	NR	• Have been diagnosed with lung cancer • Have lung nodules under surveillance • Have had hemoptysis of unknown cause or unexplained weight loss of more than five kilograms in the past year • Are currently undergoing diagnostic assessment, treatment or surveillance for life-threatening conditions (e.g., a cancer with a poor prognosis) as assessed by the referring provider
Croatia: National Programme for Early Detection of Lung Cancer^50^	50–75 years	• Active smokers or those who have stopped smoking within 15 years before screening with a smoking history of at least 30 years (30 pack/years)	• Eligible regardless of comorbidities and other demographic or anamnestic characteristics	• The presence of symptoms that indicate a possible malignant disease • Having had a chest CT scan in the last 12 months • If the patient has been treated for lung cancer in the last 5 years • If the patient's general condition prevents the planned diagnostic and therapeutic procedures (eg., If people have diseases or conditions that limit potential curable lung surgery) • The patient is unable to consent to examination • The patient is unable to lie flat, hold their breath or is claustrophobic (unable to complete the LDCT)
Czechia: Early Detection Programme for Lung Cancer^51^	55–74 years	• Current or ex-smoker who have smoked 20 and more pack-years	• SOLACE program is supporting the identification of subgroups with substantially higher risk of lung cancer which include patients with COPD, fibrotic ILD and cancer survivors at high risk of lung cancer as a secondary malignancy	NR
England: National Targeted Lung Cancer Screening Programme^38^	55–74 years 364 days of age	• Have ever smoked • Risk is assessed based on smoking history and additional factors • PLCOM2012 risk of ≥1.51% over six years or LLPver2 five-year risk of ≥2.5%	• Registered with a GP practice in England • Willing and able to undergo LDCT	• Weight or physical size exceeds restrictions for scanner (e.g. >200 kg) • Participant unable to lie flat • Poor physical fitness such that treatment with curative intent would be contraindicated • Participants who have had a full thoracic CT scan in the last 12 months
Poland: National Program of Early Lung Cancer Detection^53^	50–74 years	• Smoke addictively or you have recently quit smoking • Protocol calls for 55–74 with 20 pack-years and a period of abstinence no longer than 15 years, or 50–74 with same smoking history and an additional risk factor	• Due to profession, exposer to silica, beryllium, nickel, chromium, cadmium, asbestos, arsenic compounds, diesel exhaust fumes, smoke from hard coal combustion, soot (e.g. steel workers, workers in the electronics industry and power plants, and miners) • Exposer to radon • Diagnosis of lung cancer, lymphoma, head and neck cancer or tobacco-related cancers, e.g. bladder cancer • Someone in the immediate family (first degree) has had lung cancer • Diagnosed with chronic obstructive pulmonary disease (COPD) or pulmonary fibrosis (IPF) • No symptoms of lung cancer and the participants general condition (WHO/ECOG/Zubrod) or accompanying diseases do not exclude invasive diagnostics and surgical procedures	NR
Republic of Korea: Korean National Lung Cancer Screening Program^54^	54–74 years	• Current smokers with a smoking history of more than 30 pack-years	NR	NR
Taiwan: National Lung Cancer Screening Program^55^	50–74 years unless eligibility is due to family history risk factor than males aged 50–74 years or females aged 45–74 years.	• Smoking history of more than 30 pack-years • Are willing to quit smoking (if currently smoking should agree to receive smoking cessation services) • Have quit smoking for less than 15 years	• Can be eligible due to having any of the following two lung cancer risk factors: 1) family history of lung cancer (parents, children or siblings have been diagnosed with lung cancer or 2) heavy smoker • Have health insurance status	• Pregnancy • Have had chest CT within the past 12 months • Have been diagnosed with lung cancer previously • Are unable to undergo thoracentesis or surgery • Hemoptysis of unknown cause • Chest x-ray with the past month shows suspicious lung cancer lesions • Unexplained weight loss of more than 6 kg in the past year
UAE–Emirate of Abu Dhabi: Department of Health Lung Cancer Screening Program^56^	55–75 years	• 30 pack-year history of smoking and/or tobacco cessation less than 15 years • 20 pack-year history of smoking and/or tobacco cessation less than 15 years and one additional risk factor (Include cancer history, lung disease history as chronic obstructive pulmonary disease or pulmonary fibrosis, family history of lung cancer, radon exposure and occupational exposure of silica, cadmium, asbestos, arsenic, beryllium, chromium, diesel fumes, and nickel) • 20 year history of water pipe (shisha) and/or dokha, medwakh, and/or all other forms of smoked tobacco use	NR	• Metallic implants or devices in the chest or back, such as pacemakers or Harrington fixation rods • Patient history of lung cancer • Requirement for home oxygen supplementation • Unexplained weight loss of >7 kg in the 12 months prior • Pneumonia or acute respiratory infection treated with antibiotics in the 12 weeks prior • Chest CT examination in the 12 months prior • Patient not considered a good candidate for surgical treatment
USA: National Lung Cancer Screening Program^57^	50–80 years	• 20 pack-year smoking history and currently smoke or have quit within the past 15 years	• NR	• NR

Note. The language presented in this table is largely derived from, and consistent with, that from the policy documents referenced, NR–Not reported.

Eight (73%) countries utilised pack-years to measure smoking intensity, with five countries (45% [Australia, Croatia, Emirate of Abu Dhabi, Republic of Korea, Taiwan]) adopting 30-pack-years as an eligibility requirement. The remaining three programs (27% [British Columbia, England, Ontario]) utilised either the PLCOm2012^58^ or LLPv2^59^ risk prediction models to determine eligibility. For some programs, context-specific criteria, were also applied (e.g. the Emirate of Abu Dhabi included water pipes).^56^ Most countries listed criteria excluding participation in their respective programs. Common exclusion criteria included presenting with symptoms of lung cancer, inability to participate in a CT scan, having had a CT scan in the 12 months prior, and having been previously treated for lung cancer. For some programs, fitness to participate in curative lung cancer treatments was required (Croatia, Emirate of Abu Dhabi, Taiwan).

### Estimates of eligible current smoking populations

Data collected through the ITC Project allowed for demographics and a measure of pack-years to be estimated for Australia, Canada, England, the US, the Republic of Korea and Poland. The proportion meeting the risk thresholds for programs utilising the PLCOm2012^58^ or LLPv2^59^ were utilised for Canada and England. Applying the relevant criteria, estimates of eligibility were estimated and analysed for Australia, Canada (British Columbia and Ontario), Croatia, Czechia, England, the US, the Republic of Korea and Poland.

It was estimated that the fewest people who currently smoked met eligibility criteria for programs in the Republic of Korea (10.7%), England (16%), and Australia (16.5%). Each of these countries differed in their eligibility requirements for people who currently smoked, with the only similarity being the 30-pack-year requirement for both the Republic of Korea and Australia. Eligibility for the current smoking populations in remaining countries ranged from 19.4% (Ontario, Canada) to 27.8% (US).

When estimating the proportion of the total adult population made up by eligible people who currently smoke, Australia, England, and the Republic of Korea’s programs were estimated to be the most narrowly targeted, with eligible people who currently smoke comprising of less than 2% of the adult population. These countries were closely followed by Ontario, Canada (2.1%) and British Columbia, Canada (2.5%). Proportions exceeded 5% in Czechia, Croatia, and Poland, with Croatia having the largest estimated reach in 6.7% of Croatia’s adult population.

Table 3 represents the estimated proportion of people who currently smoke within population groups eligible to participate in 2022.

**Table 3:** Eligibility estimates for people who currently smoke by program.

Country	Criteria for modelling		% of people who currently smoke eligible%, [ 95% CI]	% of total adult (18+) population eligible% [95% CI]
	Age range	Smoking/Risk		
Australia	50–70 years	30 pack-years	16.48 [16.43–16.53]	1.66 [1.66–1.67]
Canada: British Columbia	55–74 years	- Smoking history of 20 years + PLCOm2012 risk of ≥1.5% over 6 years	23.18 [23.06–23.30]	2.50 [2.50–2.51]
Canada: Ontario^a^	55–74 years	- Smoked cigarettes daily for at least 20 years (not necessarily 20 years in a row) + PLCOm2012 risk of ≥2% over 6 years	19.43 [19.36–19.50]	2.099 [2.096–2.101]
Croatia	50–75 years	30 pack-years	22.61 [22.52–22.69]	6.65 [6.64–6.65]
Czechia	55–74 years	20 and more pack-years	24.75 [24.69–24.81]	5.79 [5.79–5.80]
England	55–74 years	Have ever smoked + PLCOM2012 risk of ≥1.51% over six years or LLPver2 five-year risk of ≥2.5%.	16.03 [16.00–16.06]	1.828 [1.826–1.829]
Poland	50–74 years	20 pack-years	23.47 [23.44–23.50]	5.069 [5.067–5.070]
Republic of Korea	54–74 years	30 pack-years	10.72 [10.69–10.74]	1.918 [1.917–1.919]
USA	50–80 years	20 pack-years	27.84 [27.83–27.86]	4.316 [4.315–4.316]

Note. Estimates are reported to two decimal places. Where this results in a flat confidence interval, three decimal places are used to retain variability.

a Estimates for Ontario reflect the eligibility age range of the program prior to its revision in 2025.^49^

## Discussion

With evidence of the beneficial impact of screening for lung cancer by LDCT scans on survival outcomes,^4^^,^^5^^,^^60^^,^^61^ an increasing number of countries have started to implement screening programs for lung cancer.^8^ This study aimed to compare the program parameters, eligibility criteria and estimated screening-eligible population of currently implemented programs for people who currently smoke. Despite many of the programs being informed by two key clinical trials (NELSON and NLST),^4^^,^^5^ countries varied in their eligibility criteria, with no two countries having identical criteria. Most countries utilised pack-years as a measure of smoking intensity, with required pack-years ranging between 20 and 30. Most commonly, those aged 50–74 years were eligible to participate in lung cancer screening, with the exception of Australia (50–70 years), which was the only program to cease at 70 years of age. Similarly, Taiwan was the only country to have a starting age below 50, where women who have a family history of lung cancer were eligible to participate from the age of 45. The US had the widest eligible age range, with their program commencing at 50 years of age (similar to Australia), with a cut-off at 80 years of age.

With respect to eligible people who currently smoke, the Republic of Korea’s program allowed the fewest people to access their program comparatively, followed by England and Australia. When estimating the proportion of the total adult population made up by eligible people who currently smoke, Australia, England, and the Republic of Korea’s programs were estimated to be the most narrowly targeted. Conversely, Croatia, Czechia and Poland, countries in the European Union, largely demonstrated greater reach with their programs. However, this may be expected as each of these European Union countries had higher current smoking rates compared to England, Australia, and Canada, reflecting substantial differences in enforcement of tobacco control and smoking cessation strategies.^62^ This consideration of higher smoking rates, combined with broader age ranges, lower pack-year thresholds, and older population compositions are likely to have contributed to greater estimated program reach in the European countries. Although the US had a lower current smoking rate than Poland, Czechia, and Croatia, a higher percentage of those currently smoking—an estimated 27.8%—were eligible for screening. This likely reflects the broader eligible age range and a lower pack-year requirement (20-pack-years) in the US.

For screening programs to achieve their intended impact, it is essential that a sufficient proportion of eligible individuals participate.^9^ Lung cancer screening aims to target those who are at a high risk of developing lung cancer, with those at low risk considered not suitable due to the potential harms of false positives, overdiagnosis, and radiation-related lung cancer death.^63^^,^^64^ An important element of this is the use of pack-years as opposed to a risk prediction tool to assist in identifying those who would benefit from screening. Programs that used either pack-years or a risk prediction tool were included in this review, with most countries adopting pack-years. The International Lung Screening Trial (ILST)^65^ is currently underway, to allow for comparison between the predictive accuracy of two commonly used screening tools to enter the program: the Prostate, Lung, Colorectal, and Ovarian model (PLCOm2012)^58^ and the United States Preventive Services Task Force (USPSTF) screening selection criteria.^57^ Findings will be a key part of understanding the balance between sensitivity, specificity, positive and negative predictive values to inform eligibility criteria for participation in a lung cancer screening program. Interim results suggest that the PLCOm2012 may be more efficient at identifying those who would benefit from lung cancer screening.^39^ Cost-effectiveness analysis suggests that risk-based strategies for screening are more cost-effective than the USPSTF recommendations for screening implemented in the US.^66^ Currently, only three of the existing lung cancer screening programs use a risk prediction tool to assess eligibility. Further, there is emerging evidence that shifting from pack-years to smoking duration to assess screening eligibility may increase the sensitivity of the eligibility assessment, to appropriately expand screening of high-risk populations.^67^^,^^68^ However, changes to eligibility criteria must balance the risks and benefits of screening, the cost-effectiveness of expanding a program and acceptability amongst clinicians, who are integral to facilitating access to lung cancer screening.^63^ As different programs develop and revise their eligibility criteria, consideration should be given to the proportion of the at-risk population that are eligible to participate, and to how this impacts cost-effectiveness of screening programs. Given criteria that are more complex are less likely to be followed in practice,^69^ consideration should be given ensuring eligibility criteria can be applied in practice, maximising feasibility, engagement from clinicians and uptake of screening by those eligible, such as the example of shifting eligibility criteria to duration rather than pack-years which may be more simple to apply to at-risk populations. A further consideration is the availability of risk prediction tools that are validated and fit-for-purpose for all population groups, to support equitable access.

Given the uptake of lung cancer screening has been low amongst some population groups, it is important that programs are evaluated. The US have previously reviewed their program, adjusting the eligible age range and reducing the number of pack-years required, broadening the reach of their program.^57^ Similarly, in 2025, Ontario broadened their eligible age range, increasing from 55-74 years to 55–80 years. The inclusion of evaluation is crucial, as programs seek to optimise how they operate, and maximise the benefits of screening and early diagnosis where possible. We recommend that future research and evaluation should continue to review existing programs to ensure that they are responsive to optimise engagement and cost-effectiveness.

In assessing program parameters, consideration was given to programs that documented strategies for engaging key population groups in lung cancer screening, with only three programs having clear strategies (Australia, Canada—British Columbia, and Ontario). Consideration of equitable access across key population groups should remain a priority, and is a direction for future research, with few countries developing strategies for equitable access. Screening rates have been demonstrated as being lower in some populations, including American Indians and Alaska Natives.^14^ Additionally, those living in rural and remote areas face access barriers to cancer prevention and care,^70^ including lung cancer screening programs.^71^ Targeted efforts addressing this, such as the lung cancer screening trucks or buses in Australia^45^ and England,^40^ and engagement with appropriate organisations to improve cultural safety and acceptability of programs, as done in Canada^72^ and Australia,^73^, ^74^, ^75^ are an important part of delivering equitable and accessible screening programs. Reducing these inequities should be a priority, incorporating the views of clinicians who facilitate access to lung cancer screening programs, and populations who experience these disparities are essential to developing appropriate strategies.

### Strengths and limitations

To our knowledge, this is the first international comparison of lung cancer screening program eligible populations, providing evidence of the variation in program eligibility criteria, implementation and execution. A structured and exhaustive search strategy was applied for all included programs to identify country-specific grey literature. Where there were missing data, relevant contacts listed on a country’s primary resource were contacted for assistance in obtaining further information. Where no further information was available, or making contact was unsuccessful, country-specific grey literature resources were supplemented by peer-reviewed academic literature. Tobacco use data were sourced from a large longitudinal cohort study, ensuring comparability across countries. In this analysis, eligibility for the screening program was estimated only for people who currently smoke. Individuals who have quit smoking but may still meet eligibility criteria were not included in the model. As such, the estimated proportions—expressed relative to both the total adult population and the current smoking population—reflect only the subset of eligible individuals of which currently smoke. These estimates do not represent the full population that are eligible for screening. This is also true for the Ontario screening program, for which modelling was conducted using the age range corresponding to the population that the risk prediction tool was originally applied (55–74 years),^40^ and therefore the estimate does not reflect the full currently eligible population (55–80 years). The measure of years smoked adopted an assumed initial age of 18 years old. As age of initiation has increased over time in some populations,^41^ those within the eligible age ranges outlined for lung cancer screening may have commenced smoking prior to the age of 18. Therefore, the years smoked variable included in the model is likely to be a conservative estimate. Additionally, measures of current smoking were utilised for modelling and may not reflect lifetime smoking behaviours. Due to the lack of comparable tobacco use data, modelling could not be completed for all countries included in the policy landscape analysis. A further limitation is that as countries develop programs, or adjust program parameters, there may be countries that would now be recognized as having national, organized screening programs that were not included in this analysis, with China being a key example.^76^ The definition of an active lung cancer screening program is complex, and while we chose to include countries that were recognised as meeting the inclusion criteria described in the methods, it is important to acknowledge that countries across the Western Pacific region, and internationally, are at various stages of adoption; with policy in development, clinical trials underway and implementation studies in progress. Across the Western Pacific, China, Japan, Singapore, New Zealand, Hong Kong and Malaysia are at various, and changing, stages of introducing active lung cancer screening programs.^77^ While China has developed national guidelines^78^ and implemented large scale lung cancer screening initiatives,^79^ according to the Lung Cancer Policy Network interactive map, it does not have an active program and was not included in these analyses.^77^ This is an example of the continuously evolving landscape, reflecting the development of local evidence, policies and funding that support screening programs. In the context of changing and adjusting programs, this study still provides a useful international comparison.

## Conclusion

Lung cancer screening programs play a significant role in supporting the early detection of lung cancer to improve lung cancer outcomes both locally and globally. Unlike other population screening programs, they are unique in their risk-based approach to eligibility. Currently, there is substantial variation in the proportion of people who currently smoke and are considered eligible to participate, and in the parameters of existing programs internationally. This work demonstrated that approaches to assessing risk and eligibility are interpreted and implemented differently, impacting the size of eligible populations in each country. Given the targeted nature of these programs, it is imperative that programs incorporate strategies to ensure participation by all at-risk groups, especially those historically underrepresented in other screening programs.

## Contributors

HJ: Conceptualization; Data curation; Formal analysis; Project administration; Writing–original draft; Writing–review & editing, LG: Conceptualization; Formal analysis; Supervision; Writing–review & editing, PML: Conceptualization; Supervision; Writing–review & editing, VMW: Conceptualization; Supervision; Writing–review & editing, VM: Conceptualization; Supervision; Writing–review & editing, AU: Conceptualization; Supervision; Writing–review & editing, HHY: Data curation; Formal analysis; Writing–review & editing, CG: Data curation; Writing–review & editing, GTF: Data curation; Writing–review & editing, AH: Data curation; Writing–review & editing, KAE: Data curation; Writing–review & editing, KP: Data curation; Writing–review & editing, HGS: Data curation; Writing–review & editing & YK: Writing–review & editing.

## Data sharing statement

In each country participating in the International Tobacco Control Policy Evaluation (ITC) Project, the data are jointly owned by the lead researcher(s) in that country and the ITC Project at the University of Waterloo. Data from the ITC Project are available to approved researchers 2 years after the date of issuance of cleaned data sets by the ITC Data Management Centre. Researchers interested in using ITC data are required to apply for approval by submitting an International Tobacco Control Data Repository (ITCDR) request application and subsequently to sign an ITCDR Data Usage Agreement. The criteria for data usage approval and the contents of the Data Usage Agreement are described online (http://www.itcproject.org).

## Declaration of interests

HJ reports consulting fees from Cancer Australia and support to attend meetings from the International Association for the Study of Lung Cancer. GTF has been an expert witness or consultant for governments defending their country’s policies or regulations in litigation and reports grant funding from the Ontario Institute for Cancer Research, and honorariums and travel support from US National Association of Attorneys General. CG has served as an expert witness for the Australian Government in litigation and reports grant funding from World Health Organization and Department of Health, Disability and Ageing, support to attend meetings from Department of Health, Disability and Ageing, NSW Government and National Health and Medical Research Council (NHMRC), membership to Society for Research on Nicotine and Tobacco and Project Sunset, and editor roles with Tobacco Control (BMJ Journals) and Tobacco Prevention and Cessation. AH reports grant funding on behalf of their employer from the NIH, FAMRI, and the New York State Department of Health, consulting and/or speaker fees from academic research organisations including the NCI, UCSF, and the U Minnesota. KE reports consulting fees from UKRI consulting and support to attend meetings from the Society for the Study of Addiction and King’s College London. AU, PM and VW report consulting fees from Cancer Australia. KP reports grant funding, consulting fees, honorariums and support to attend meetings from Adamed Pharma SA, consulting fees from Aflofarm Pharma Poland and an honoraria from JNTL Consumer Health Poland. All other authors have no conflict of interests to declare.
